# Investigating habits in humans with a symmetrical outcome-revaluation task

**DOI:** 10.3758/s13428-022-01922-4

**Published:** 2022-07-22

**Authors:** P. Watson, T. E. Gladwin, A. A. C. Verhoeven, S. de Wit

**Affiliations:** 1 The Habit Lab, Department of Clinical Psychology, University of Amsterdam, Amsterdam, Netherlands; 2 Amsterdam Brain and Cognition, University of Amsterdam, Nieuwe Achtergracht 129B, 1018 Amsterdam, WS Netherlands; 3 School of Psychology, UNSW Sydney, Sydney, Australia; 4 Institute for Lifecourse Development, University of Greenwich, London, UK; 5 Behavioural Science Institute, Radboud University Nijmegen, Nijmegen, The Netherlands

**Keywords:** Habits, Outcome devaluation, Goal-directed control, Symmetrical outcome-revaluation task, Associative learning

## Abstract

The translation of the outcome-devaluation paradigm to study habit in humans has yielded interesting insights but proven to be challenging. We present a novel, outcome-revaluation task with a symmetrical design, in the sense that half of the available outcomes are always valuable and the other half not-valuable. In the present studies, during the instrumental learning phase, participants learned to respond (Go) to certain stimuli to collect valuable outcomes (and points) while refraining to respond (NoGo) to stimuli signaling not-valuable outcomes. Half of the stimuli were short-trained, while the other half were long-trained. Subsequently, in the test phase, the signaled outcomes were either value-congruent with training (still-valuable and still-not-valuable), or value-incongruent (devalued and upvalued). The change in outcome value on value-incongruent trials meant that participants had to flexibly adjust their behavior. At the end of the training phase, participants completed the self-report behavioral automaticity index – providing an automaticity score for each stimulus-response association. We conducted two experiments using this task, that both provided evidence for stimulus-driven habits as reflected in poorer performance on devalued and upvalued trials relative to still-not-valuable trials and still-valuable trials, respectively. While self-reported automaticity increased with longer training, behavioral flexibility was not affected. After extended training (Experiment 2), higher levels of self-reported automaticity when responding to stimuli signaling valuable outcomes were related to more ‘slips of action’ when the associated outcome was subsequently devalued. We conclude that the symmetrical outcome-revaluation task provides a promising paradigm for the experimental investigation of habits in humans.

## Introduction

Habits are a major topic of investigation by psychologists, and different measures have been developed to capture habit formation and investigate its determinants (Verhoeven & de Wit, 2018). Under investigation in the current study are learnt S-R habits, as defined by associative dual-process models of behavior (de Wit & Dickinson, 2009; Dickinson, 1985). Under this framework, habits are defined as responses (R) that are triggered by environmental stimuli (S) and that are ‘behaviorally autonomous’ of the current desirability of their outcome (de Wit & Dickinson, 2009; Dickinson, 1994, 2016). In contrast, goal-directed actions are mediated by anticipation and evaluation of the outcome (Heyes & Dickinson, 1990), and can therefore be flexibly adapted when the desirability of that outcome changes. In the field of associative learning, animal researchers have developed the outcome-revaluation paradigm to study the balance between habitual and goal-directed control (Dickinson, 1985). In more recent years, efforts have been made to translate this task to study human decision-making, but this has proven to be challenging. Here, we present a novel outcome-revaluation paradigm that has some advantages over previous designs, allowing for the experimental investigation of S-R habits in humans.

The theoretical framework for outcome-revaluation research is derived from dual-process theories, according to which instrumental behavior is under the control of habitual and goal-directed processes, with the former gaining dominant control with extensive behavioral repetition in a stable context. To illustrate, imagine that for many years you drive the same route to your office. Here, the advantage of habit formation may be that the behavior can be performed efficiently without having to actively focus on the goal. On the other hand, when one’s goals change, habits can cause ‘slips of action’, responses that are triggered by environmental stimuli independently of one’s current motivation. For example, you may intend to drive to your new office but when approaching a crucial crossroads, you accidently turn left along the route that you always used to drive to the old office.

To determine the habitual or goal-directed status of behavior in a highly controlled experimental setting, animal researchers have developed the outcome-revaluation paradigm (Adams & Dickinson, 1981; Dickinson, 1985). Typically, instrumental training (e.g., lever press → food) is followed by devaluation of the outcome (through e.g., satiation), meaning that it is no longer desirable. Subsequently, responding for the outcome is assessed in extinction (i.e., without any reinforcement, to prevent further learning). If responding for the devalued outcome is immediately reduced, then behavior is argued to be mediated by anticipation and evaluation of the outcome, and thus to be goal-directed. In contrast, persistent responding for the devalued outcome suggests that responses are triggered by the context in a habitual stimulus-response (S-R) manner. Using this paradigm, animals have been shown to be capable of flexible, goal-directed action, but after extensive training their behavior becomes habitual (Adams, 1982; Adams & Dickinson, 1981; Dickinson, 1985).

In recent years, the outcome-revaluation paradigm has been translated to the study of the role of habits in human decision-making. One of the most commonly used outcome-devaluation tasks in human research is the slips-of-action task (de Wit et al., 2012; Gillan et al., 2011). In the initial instrumental discrimination learning phase, participants learn the relationships between stimuli, responses, and outcomes (typically pictures of fruit that are worth points; e.g., bananas signal that a right key press will earn a coconut outcome). These discriminations could be learned in a goal-directed manner by encoding the outcomes as part of the associative structures controlling performance (bananas-coconut-right) or by establishing simple S-R associations (bananas-right). Dual-process models of action control propose that both associative structures can be encoded during learning and support flexible goal-directed actions or habitual responses, respectively. To directly investigate the balance between goal-directed and habitual control, some of the outcomes are subsequently devalued through instruction. Continued responding for those outcomes will lead to a loss of points. Participants are then presented with the stimuli and must decide, under considerable time pressure, whether to respond for the signaled outcome. Commission errors (responding for devalued outcomes), or ‘slips of action’, are interpreted as evidence for habitual control.

Using the slips-of-action task, individual differences in the tendency to rely on inflexible, habitual responding have been related to impulsive-compulsive behavior and its neurobiological basis in both patient populations and the general population (see for reviews: Verhoeven & de Wit, 2018; Watson & de Wit, 2018). There are some key features of the slips-of-action task design that encourage the formation and expression of S-R associations. The use of discriminative stimuli during training supports the formation of distinct S-R associations (rather than concurrent instrumental training where multiple responses are available and the context becomes a general cue associated with all possible responses; Colwill, 1994; Thrailkill et al., 2018; Vandaele et al., 2017). Furthermore, forcing participants to respond under time pressure reduces the likelihood that participants will be able to inhibit the tendency to respond in an S-R manner for devalued outcomes (Hardwick et al., 2019). These features of the slips-of-action task were also included in the novel, *symmetrical* outcome-revaluation task introduced here.

However, there are some disadvantages to the slips-of-action task, which the *symmetrical* outcome-revaluation task improves upon. The main issue with the former task is that during the instrumental training phase all outcomes are valuable, so participants should respond to every stimulus. As such, the specific fruit outcomes may not function as goals for the participants during the training phase. Even though participants are explicitly asked to pay attention to the outcomes of their actions (e.g., to learn that bananas signal the availability of coconuts upon pressing the right key), strictly speaking they do not need to learn about these outcomes to perform well according to the S-R mappings during the training phase (Buabang et al., 2021; De Houwer et al., 2018). That is, participants simply need to learn that a right key press in the presence of the bananas will earn them points. Because initially all outcomes are valuable (i.e., worth points), the specific (fruit) outcome identity becomes irrelevant, and knowledge of the correct S-R associations is sufficient to successfully complete training. Therefore, individual differences in test performance and demonstrations of impaired performance, for example in certain psychopathologies, may reflect partly the extent to which participants follow the task instruction to pay attention to the outcomes during training.

The most important difference in the novel symmetrical outcome-revaluation task is that only half of the outcomes are valuable and should be collected during each training block. This symmetrical design ensures that participants initially learn to perform the responses in a goal-directed manner based on the current value of the signaled outcome, before potentially transitioning to relying on S-R associations. Specifically, at the start of each training block, participants are instructed which two outcomes will lead to points, and which two will lead to deduction of points. Subsequently, at the start of each trial a discriminative stimulus is presented that signals the availability of one of four outcomes. Participants are instructed that they should only respond (i.e., press the spacebar) if the signaled outcome is valuable. Therefore, this task ensures that participants have to learn the identities of the signaled outcomes (as a function of the current stimulus) and then decide whether or not to respond for them based on their current value (the ‘valuable’ outcomes change on each block). As such, they cannot simply rely on S-R associations from the outset. However, over the course of training, S-R relations remain stable, ensuring that S-R associations can be formed and can later compete for behavioral control.

In the subsequent test phase, some of the signaled outcomes are devalued to determine the degree to which well-learnt S-R associations can trigger inappropriate responses under time pressure. In this respect, the test phase of the symmetrical outcome-revaluation task is highly similar to that of the slips-of-action task. However, instead of comparing performance on devalued trials with still-valuable, the symmetrical design offers the advantage that we can compare performance on devalued trials with that on still-not-valuable trials. In both cases, a NoGo response is required, but only on devalued trials can learnt S-R associations interfere with performance. Furthermore, we can study a similar contrast on Go test trials where the outcome is valuable. Performance on still-valuable trials, where participants can rely on learnt S-R associations, is contrasted with performance on upvalued trials, that also require a Go response but lack this benefit of S-R learning. Therefore, during the test phase, outcome value is congruent with that during training on half of the (Go and NoGo) trials (still-valuable and still-not-valuable) and incongruent (devalued and upvalued) on the other half.

In the present studies, we also investigated the effect of behavioral repetition on test performance. Despite several attempts, experimental investigations of habit formation in humans have failed to provide convincing evidence for the development of behavioral autonomy as a function of behavioral repetition during training (de Wit et al., 2018; Pool et al., 2021; Tricomi et al., 2009). Although there are recent suggestions that participants are slower to make novel responses in the presence of stimuli that have been overtrained with a different response (Hardwick et al., 2019; Luque et al., 2020), participants rarely make more overt ‘slips of action’ (commission errors) for long-trained relative to short-trained devalued outcomes, in classical outcome devaluation tasks (de Wit et al., 2018). To investigate whether performance on the novel symmetrical outcome-revaluation paradigm is affected by training length, we included a within-subject overtraining manipulation such that during training, some stimuli were shown (and responded to) more frequently than others. Poorer performance on trials where participants had to refrain from making a previously learned response (i.e., devalued trials relative to still-not-valuable) or generate a response when one had not previously been required (i.e. upvalued relative to still-valuable), following long relative to short training would therefore be indicative of stronger S-R associations developing as a consequence of more extensive training.

Finally, we investigated how performance on the symmetrical outcome-revaluation task relates to a self-report measure of automaticity. While the outcome-revaluation task is considered the canonical assay of habits in the field of associative learning, the fields of social and health psychology have mainly used self-reported measures to study the habit status of real-life behaviors (Verhoeven & de Wit, 2018), including teeth-flossing, unhealthy snacking (Verhoeven et al., 2012), exercise (review: Gardner et al., 2011; Ouellette & Wood, 1998) and consumer behavior (Labrecque et al., 2017). The most commonly used self-report measurement is the 12-item self-report habit index (SRHI: Verplanken & Orbell, 2003). Particularly in the domain of health behaviors, scores on the SRHI have been related to the frequency with which certain behaviors are carried out (e.g., self-reported fruit consumption) or to more objective measurements such as the choice between a healthy versus unhealthy snack when offered in a lab setting (see Gardner et al., 2011 for a meta-analysis of these studies). The self-report behavioral automaticity index (SRBAI), a shortened version of the SRHI consisting of just four automaticity-related items, has been shown to have comparable predictive utility (Gardner et al., 2012). However, self-report measures have been criticized for relying on participant insight into what is argued to be relatively automatic behavior (Gardner & Tang, 2014; Hagger et al., 2015; Sniehotta & Presseau, 2012). The question arises to what extent these self-report measures relate to inflexible performance on outcome-revaluation tasks. One previous study reported a modest increase in self-reported automaticity as a result of prolonged training on the slips-of-action task but did not relate self-reported automaticity to slips-of-action frequency (de Wit et al., 2018). In the current study, we sought to investigate this relationship more directly, by comparing self-reported automaticity of S-R learning following short versus long instrumental training and determining whether automaticity of Go responses was predictive of action slips in the presence of stimuli signaling devalued outcomes.

To conclude, in the current study we conducted two experiments with the novel symmetrical outcome-revaluation task. We expected that learnt S-R associations would trigger an inappropriate response on devalued trials (relative to still-not-valuable). Likewise, we expected to observe the benefit of S-R learning on still-valuable trials where participants were consistently required to make a response to earn a valuable outcome across training and test (relative to upvalued). Furthermore, we investigated whether increasing the number of behavioral repetitions (i.e., extended training) would reduce flexible goal-directed control. Firstly, in Experiment 1, some stimuli were presented three times more frequently than others. We also investigated whether self-reported automaticity of responding during training was predictive of outcome-revaluation test performance.

## Experiment 1

### Methods

All measures and manipulations (in both experiments) are disclosed in the present manuscript.

#### Participants

We aimed to test at least 30 people during a fixed period of time, based on pre-agreed availability of lab space and the experimenter. Thirty-six participants were recruited from the University of Amsterdam participant website over a 2-month period. Advertisements stated that it was a fun study where participants would have to earn as many points as possible. Eight participants were excluded from all analyses (see results). The remaining 28 participants (24 female, four male) had a mean age of 22.4 years (SD 3.6 years). Using G*power (Faul et al., 2007) we conducted a sensitivity analysis for repeated measures ANOVA of the crucial test phase data (eight trial types, with mean correlation between measures of 0.26) with alpha of 0.05, power of 0.8 and a total sample size of 28. This analysis indicated sufficient power to detect a small-medium effect (*f* = 0.22). Participants received either €10 or course participation credits for taking part. The Psychology Ethics Committee of the University of Amsterdam approved the study.

#### Stimuli and materials

##### Symmetrical outcome-revaluation task (SORT)

**Fig. 1:**
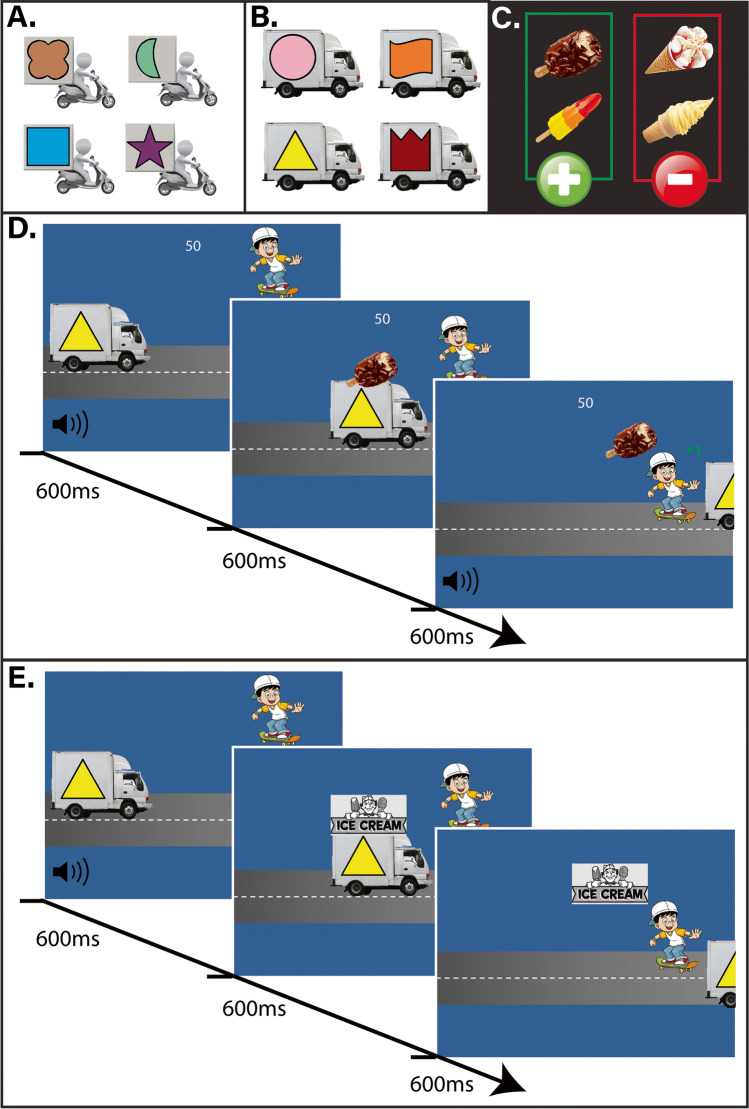
Stimuli and task procedure: **A** the four van stimuli; **B** the four scooter stimuli; **C** the outcome value instruction screen shown at the beginning of each block. Participants should only collect the ice creams depicted in the green box; **D** trial structure for the training trials. Participants had to decide whether to respond (or not) in the first 600 ms, before the image of the ice cream was displayed on the moving van (duration 600 ms). If participants did respond in time, they collected the ice cream as the vehicle left the screen and received feedback for 600 ms (see text for details); **E** trial structure for the test phase. The ice creams were no longer depicted, and no feedback was given

This computerized paradigm was programmed in Presentation (version 18.1) and consisted of an instrumental training phase followed by an outcome-revaluation test phase during which outcome values were either congruent with training value or incongruent (i.e., devalued or upvalued). The total duration was approximately 45 min. The task design is depicted in Fig. 1 and described in the procedure section. The study made use of various images (see Fig. 1a–c): the instrumental outcomes were four different ice creams – a Cornetto, a Magnum, a Rocket, and a soft serve ice cream (in a cone). The discriminative stimuli consisted of four scooters and four vans, each with a different abstract colored logo.

##### N-back task

As a measure of working memory the N-back task was used, based on that of Jaeggi and colleagues (Jaeggi et al., 2010). The stimuli were eight yellow, novel shapes, presented in a random sequence, each for 500 ms with 2500 ms between each shape presentation. Participants could push the spacebar during this 3-s window to indicate that the current stimulus was a target (i.e., had appeared either two or three iterations earlier). The task consisted of six blocks (three blocks each of 2-back and 3-back) each consisting of 20 trials in which participants could make a response. The task began with a short demo phase (ten trials of each level). The score was calculated as (total number of hits – total false alarms)/ total number of blocks. A higher score thus indicates higher working memory capacity.

##### SRBAI

The SRBAI (Gardner et al., 2012) is a four-item scale in which participants are asked to reflect on the degree to which a behavior begins “before I realize I am doing it”, is done “automatically”, “without having to consciously remember” and “without thinking”. For each item participants rate on a scale from 1 “strongly disagree” to 7 “strongly agree”. The mean score is then calculated. The SRBAI has been found to have good reliability and validity (Gardner et al., 2012). We adapted the SRBAI to measure automaticity at the end of the learning phase. To this end, a short questionnaire was programmed in Inquisit. On each screen of the questionnaire, participants saw the four scooters. Next to each stimulus was the question “what was the correct response for this vehicle?” with radio buttons labelled as “respond” and “not respond”. For each scooter stimulus participants were instructed to respond on a VAS scale ranging from 1 (completely disagree) to 7 (completely agree) to answer a question from the SRBAI e.g., “This response (responding or not responding) was something I did automatically”. After completion of the first question, they then clicked through to answer the remaining items of the SRBAI and repeated this process for the van stimuli. Cronbach’s alpha was calculated separately for the eight items that pertained to each stimulus type. The mean Cronbach’s alpha was 0.87 indicating good reliability of the SRBAI.

#### Procedure

The experiment took place in a plain lab room. Participants were told that they were going to play the “Sneaky Skateboard Game” where they were playing as a skateboarder and had to collect as many ice creams (and points) as possible. The participant with the most points at the end of the study received a pair of cinema tickets. All participants started with 50 points.

##### Instrumental training phase

Prior to the instrumental training phase, participants were told that they would first be shown which ice creams should be collected to earn points and which ice creams should not be collected as they would lead to the deduction of points. They were told that different vans and scooters were carrying the ice creams and that they would have to learn by trial and error which vehicle was carrying which ice cream. They were instructed that the vehicles would move fast, so if they wanted to collect the ice cream that the vehicle was carrying, they had to push the spacebar as quickly as possible once the vehicle appeared. Participants first performed a short 1-block demonstration of the training phase, with different outcomes (i.e., pizza slices) and stimuli (i.e., different vehicles and logos) than in the real task.

After the demonstration, the real training phase started. Each block of instrumental training began with an *outcome value instruction screen* in which the four ice creams were shown: two were highlighted in green (the valuable ice creams) and two were highlighted in red (the not-valuable ice creams; Fig. 1c). After four seconds, this screen was replaced with the *outcome value memory test*. Here, they were shown the four ice creams (randomized order) and using the mouse, they had to select which of the two ice creams would be valuable in the subsequent block. If they made an error, they were shown the outcome value instruction screen again until they were able to accurately complete the outcome value memory test. This was to ensure that any failures to collect/not collect ice creams were not simply due to participants not encoding the value of the ice creams. The trial sequence then began. The current total score was constantly presented at the center top of the screen. On each trial, a stimulus (either a van or a scooter with a particular abstract logo) appeared on the left-hand side of the screen (Fig. 1d). The representation of the participant, a cartoon skater, was presented at the top right of the screen. A ‘boing’ sound played to signal the start of the trial and the vehicle stimulus began to move from left to right. After 600 ms (when the vehicle was halfway across the screen), the ice cream associated with that particular stimulus appeared on top of the vehicle (Fig. 1d). Importantly, participants had to make a choice as to whether or not to respond on the basis of the current stimulus before the ice cream appeared. If the participant pushed the spacebar within the initial 600 ms, before the ice cream appeared, the skateboard figure moved down in time to intercept the vehicle as it approached the right-hand side of the screen. The vehicle then moved on leaving the ice cream behind with the skater, and passed off screen at 1200 ms. If the ice cream was valuable, feedback was provided in the form of a “win” sound, a green “+1” appearing and 1 point being added to the total points (displayed in the middle of the screen; see Fig. 1d). If the ice cream was non-valuable, the feedback was a buzzer sound and a red “-1” and one point was subtracted from their total score. The feedback was shown until 600 ms after the vehicle had passed off screen. If participants responded too late (after the initial 600 ms), the skateboarder moved down to the final position but after the vehicle (and ice cream) had passed the intercept point. If participants did not respond or responded too late, then the vehicle and its associated ice cream simply traveled across the screen, and both passed off screen at 1200 ms. In these latter two cases, no extra feedback was given and the final screen (containing the skateboarder but no ice cream) simply paused for 600 ms.

Each block consisted of 16 trials in which either the four scooters or the four vans were presented. In order to manipulate the amount of exposure to the S-R-O contingencies during training, two of these van/scooter stimuli in each block (one associated with a valuable and one with a not valuable ice cream) were each seen six times, whereas the other two stimuli were each shown only twice (see Table 1). In other words, the extensively trained stimuli were presented three times as often as the moderately trained stimuli. Stimulus order was randomized. At the end of each block participants saw a screen with information on their performance, e.g.: *“You successfully collected 10 Magnums and Cornettos. You responded three times incorrectly. Your score is currently 67 points”.*

**Table 1:** Trial types of the symmetrical outcome-revaluation task

Training blocks (alternating)					Test Block 1 “Collect O1 and O2”	Test trial type
Van Block “Collect O1 and O2”	S1	Valuable	O1	Short	Valuable (congruent)	Still valuable (short)
	S2	Valuable	O2	Long	Valuable (congruent)	Still valuable (long)
	S3	Not valuable	O3	Short	Not valuable (congruent)	Still not valuable (short)
	S4	Not valuable	O4	Long	Not valuable (congruent)	Still not valuable (long)
Scooter Block “Collect O3 & O4”	S5	Not valuable	O1	Short	Valuable (incongruent)	Upvalued (short)
	S6	Not valuable	O2	Long	Valuable (incongruent)	Upvalued (long)
	S7	Valuable	O3	Short	Not valuable (incongruent)	Devalued (short)
	S8	Valuable	O4	Long	Not valuable (incongruent)	Devalued (long)

During training blocks, participants saw S1-S4 or S5-S8 during separate blocks (with stimuli presented in random order in each block). The outcome (O1-O4) associated with each stimulus was consistently assigned as a valuable or not valuable outcome. Participants learned by trial and error to press the spacebar upon seeing stimuli that signaled the availability of a valuable outcome (worth 1 point), allowing for the gradual building up of S-R associations (e.g.: S1: GO → O1). Responding for not-valuable outcomes was punished by subtraction of points (minus 1 point). During the critical test phase, all eight stimuli were presented intermixed during each test block. For half of the stimuli, the associated outcome had changed in value relative to training – either upvalued or devalued. Shown in this table is an example of the contingencies in the first of eight test blocks (with different combinations of outcomes marked as valuable). We measured the ability to refrain from responding towards devalued outcomes (and increase responding for upvalued outcomes) during the test phase

They then completed a *test of S-O knowledge*. On each trial of the S-O test they saw one of the vehicles that had functioned as a discriminative stimulus during that block along with each of the four ice creams with the text: *“select the ice cream that belongs to each vehicle*”. When the participant clicked on one of the ice creams, a 10-cm-long slider appeared ranging from red to green with the caption: *“How sure are you? Left is unsure, right is sure”.* Participants had to move the mouse and click on the appropriate place. They were then shown the next three stimuli from that set. When the S-O test was completed, the next block of training began.

The blocks alternated between van and scooter blocks. Across participants the relationships between the eight vehicles and the four ice creams were randomized. During the “van” blocks, two of the ice creams (O1 and O2; e.g., Cornetto and Rocket) were always indicated as the valuable ice creams (see Table 1) and the other two ice creams (O3 and O4; e.g., Magnum and soft serve) were always indicated as the non-valuable ice creams. On the “scooter” blocks, the value of the sets of ice creams was swapped (O3 and O4 valuable; O1 and O2 non-valuable). Therefore, across the entire training phase, each ice cream was equally often designated as valuable or not valuable. The response assigned to each scooter/van stimulus (either respond or not respond) was consistent throughout the entire training phase allowing for the development of S-R associative links. In total, participants completed 32 blocks (16 van blocks and 16 scooter blocks), resulting in a total of 512 trials. The four short-trained stimuli were each seen 32 times and the four long-trained stimuli were each seen 96 times.

##### SRBAI

After completion of the training phase, participants were instructed to summon the experimenter. They were then asked to complete the SRBAI on a separate laptop.

##### Test phase

Participants were then told that, just like before, they would be instructed at the beginning of each block as to which ice creams they needed to collect. They were informed that it would now be more difficult because the vehicles delivering the ice creams had put an advertisement board on the roof which meant it was no longer possible to see which ice cream they were carrying. Participants were instructed that they would have to make their choices based on their memory of which vehicle carried which ice cream from the previous phase of the game. Furthermore, they would not see whether their responses were correct or not, but they were instructed that they were still earning (and losing) points during the test phase. Therefore, the test was conducted in nominal extinction. Participants were also instructed that during this phase of the game a mix of both vans and scooters would appear in each block. They first completed a demo block of this phase using the same pizza outcomes as during the training phase demo. The test phase was similar to the training phase - beginning with an instruction screen which informed them of which ice creams were valuable and should be collected, followed by an outcome value memory test. On each trial, a vehicle stimulus appeared on the left-hand side of the screen and the ‘boing’ sound indicated that the trial was beginning. There were, however, some crucial differences. Firstly, the identity of the ice cream outcomes was never shown during the test trials. At 600 ms after trial onset, a generic banner appeared on top of each vehicle, instead of the ice cream (see Fig. 1e). No feedback in the form of points or sounds was given during the trials, nor after each block. At the beginning of each test block, participants were instructed to collect two of the ice creams (e.g., ‘O1 and O2’). Subsequently, during each block of the test phase, all eight stimuli (both vans and scooters) were shown intermixed. Because each ice cream outcome had consistently been valuable for one type of vehicle during training (e.g., O1 was valuable in the van block; and therefore S1 became associated with a Go response) and not-valuable for the other (e.g., O1 was not-valuable in the scooter block; and therefore S5 became associated with a NoGo response), the value of the signaled outcome was congruent with training value during half of the trials (e.g., S1 trials) and incongruent during the other half (e.g., S5 trials). Specifically, this resulted in four different trial types per block (see Table 1): “still valuable” – participants had always responded in the presence of this stimulus during training and during this test block the outcome signaled by the stimulus was valuable (i.e., congruent); “still not valuable” – participants had never responded in the presence of this stimulus during training and during the current test block the outcome signaled by the stimulus was not valuable (i.e., congruent); “upvalued” – participants had never responded in the presence of this stimulus during training and during the current test block the outcome signaled by the stimulus was valuable (i.e., incongruent); and crucially, of interest, the “devalued” trials - participants had always responded in the presence of this stimulus during training and during the current test block the outcome signaled by the stimulus was not valuable (i.e., incongruent). Furthermore, each of these trial types could be broken down into short-trained (32 repetitions) and long-trained (96 repetitions), allowing us to assess the effect of amount of training on test performance. Each block consisted of each of the eight stimuli presented four times in random order (32 trials total).

The valuable ice creams during the first block were O1 and O2 (which had been valuable in the van blocks during training but not the scooter blocks). However, because all vans and scooter stimuli were now intermixed, participants had to make the incongruent, opposite response for the scooter stimuli in this block; see Table 1). During the second block, O3 and O4 were valuable which meant that the learned responses to the scooter stimuli were still correct, but to the van stimuli the participants needed to make the opposite response. The third block was a novel combination of valuable ice creams (collect O1 and O3), so that half of the van and half of the scooter stimuli were now incongruent. Similarly, in block 4 (collect O2 and O4), the other half of the van and scooter stimuli were incongruent. This meant that across the test phase the correct response for every stimulus was equally often congruent and incongruent (i.e., the same or opposite to training). The subsequent four blocks (blocks 5–8) were the same sequence as the first four blocks (256 trials in total). When participants had completed all eight blocks, they were finally asked to complete the *S-O knowledge test* again. Participants were informed as to the total points they had won.

##### N-back task

Finally, participants completed the N-back task.

#### Statistical analysis

Data analysis commenced after all data collection had been completed. For the analysis of the training data, the data were collapsed across each pair of blocks (e.g., the first block of vans was combined with the first block of scooters and so forth, reducing the 32 blocks to 16 blocks). Accuracy during the training phase (i.e., % responding, during the 600-ms response window, in order to earn valuable outcomes whilst not responding for not-valuable outcomes out of total number of trials) was analyzed using repeated measures ANOVA with block (1–16), training length of the stimulus (long-trained, short-trained) and outcome value (valuable, not valuable). For the test phase, accuracy data (% of correct responses made in the 600-ms response window) were collapsed across blocks and an ANOVA was used with within-subject variables: training length of the stimulus (long-trained, short-trained), outcome value on the current test block (valuable, not valuable) and congruence between training value and test value (congruent, incongruent). The combination of the latter two variables gives the four trial types indicated previously: still valuable, still not valuable, upvalued and devalued. All Greenhouse–Geisser *p* values are reported with the original degrees of freedom. Finally, after checking the distribution of the variables, correlational analyses were used to investigate the relationship between SRBAI scores, the N-Back task and test-phase performance. We adjusted alpha using Bonferroni correction to control for multiple correlational comparisons. Spearman’s rank-order correlations are indicated by *rho.*

### Results

To ensure that participants were attempting to complete the task correctly, we excluded anyone who had responded on less than 25% of the upvalued trials (stimuli whose associated outcome was valuable during test but had never been valuable during training). This resulted in the exclusion of eight participants, resulting in a sample size of 28 participants.

#### Training phase

**Fig. 2:**
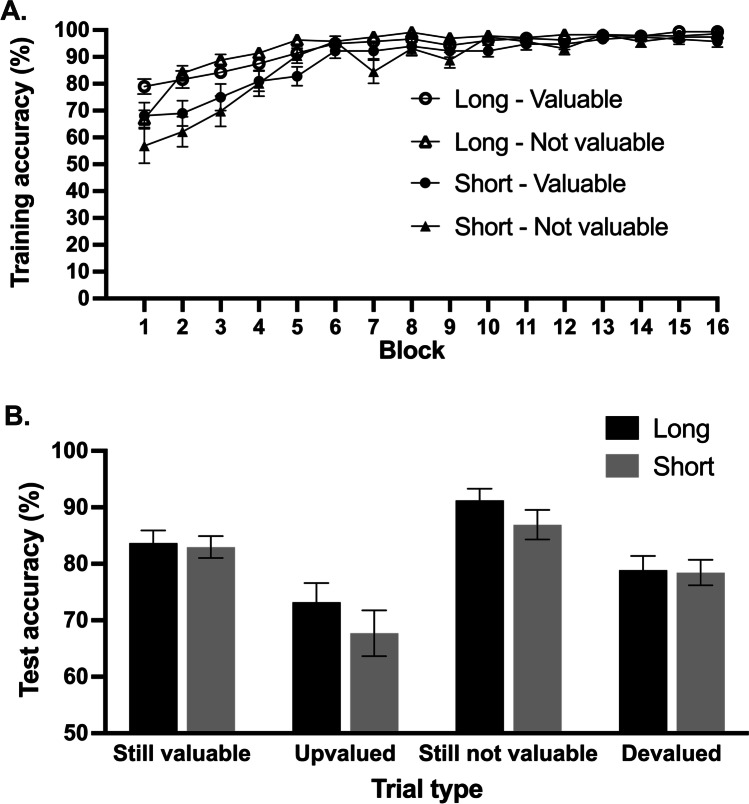
**A** Accuracy in the training phase: Participants learned across the 16 blocks of training to respond for stimuli that signaled valuable outcomes and to withhold responses for not valuable stimuli. By the final block of training there was no difference in accuracy for long-trained versus short-trained stimuli. **B** Accuracy in the test phase: Participants were less accurate on devalued trials relative to still-not-valuable trials and more accurate on still-valuable trials relative to upvalued trials. There was no significant difference in accuracy on short- relative to long-trained stimuli across trial types. *Error bars* represent within-subject standard error of the mean (Cousineau, 2005) with Morey correction (Morey, 2008)

Participants increased their accuracy over the course of training as indicated by a main effect of block, *F*(15, 405) = 62.31, *p* < 0.001, η_*p*_^2^ = 0.70 (see Fig. 2A). Overall, accuracy was higher for long-trained stimuli (main effect of long/short training, *F*(1,27) = 57.89, *p* < 0.001, η_*p*_^2^ = 0.68) but this was superseded by an interaction between block and short/long training, *F*(15, 405) = 6.4, *p* < 0.001, η_*p*_^2^ = 0.19. As can be seen in Fig. 2A, performance on trials with long-trained relative to short-trained stimuli was significantly better at Block 1, *t*(27) = 3.754, *p* = 0.001, and still at Block 9, *t*(27) = 3.03, *p* = 0.005. At Block 10, however, this difference was marginal, *t*(27) = 1.83, *p* = 0.078 and from Block 11 onwards there was no significant difference between accuracy on long and short trained stimuli: (lock 11: *t*(27) = 1.42, *p* = .167; final block: *t*(27) = 1.63, *p* = 0.114). Suggesting that there was no overall difference in accuracy for valuable relative to not-valuable stimuli in the training phase, the ANOVA did not reveal a significant main effect of stimulus value, *F*<1, *p* = 0.790, η_*p*_^2^ = 0.003. The Block X Value interaction failed to reach significance, *F*(15,405) = 2.07, *p* = 0.088, *η*_*p*_^*2*^ = 0.07, nor was there a three-way interaction (block, value, and short/long training), *F*(15,405) = 1.22, *p* = 0.280, *η*_*p*_^*2*^ = 0.04.

#### Test phase

As can be seen in Fig. 2B, learned S-R associations significantly impacted on performance during the test phase. The analysis of accuracy during the test phase revealed a main effect of value-congruency: *F* (1,27) = 25.51, *p* < 0.001, η_*p*_^2^ = 0.49. Participants were less accurate on both devalued trials (where they had to now inhibit the Go response) and upvalued trials (where a Go response was now required), relative to trials where the value of the outcome signaled by the cue was congruent with that during training. However, these effects were statistically indistinguishable for short and long training. There was no significant main effect of long/short training on accuracy rates in the test phase, *F*(1,27) = 2.57, *p =* 0.121, η_*p*_^2^ = 0.09, nor was there a significant interaction between congruence and long/short training, *F*<1, *p =* 0.967, *η*_*p*_^*2*^ = 0.00. Finally, there was a marginal trend towards higher accuracy on not-valuable (NoGo) trials than valuable (Go) trials, *F*(1,27) = 3.45, *p* = 0.074, η_*p*_^2^ = 0.11.

To examine whether individual test performance showed a consistent pattern across Go and NoGo trials, we created individual difference scores for not-valuable trials (accuracy for still-not-valuable minus devalued trials) and valuable trials (accuracy for still-valuable minus upvalued trials). Indeed, these two difference scores correlated significantly, *r*(26) = 0.556, *p* = 0.002. This may reflect that participants who showed the greatest disadvantage of stimulus-Go learning (habit formation) on NoGo trials also showed the greatest advantage on Go trials.

#### Final S-O test

As expected, participants learned and retained knowledge of the various S-O relationships. When asked to recall these after the test phase, mean accuracy was 100% on both short- and long-trained valuable (Go) trials (SDs = 0), and 97% (SD 12%) and 100% (SD 0%) on short-and long-trained not-valuable (NoGo) trials, respectively. Overall confidence was at 98% (SD 4%).

#### SRBAI

When examining the SRBAI scores as a function of stimuli repetitions (long/short training) and outcome value during training there was no significant main effect of training length, *F*<1, *p* = .350, η_*p*_^2^ = 0.03, nor of outcome value, *F*(1,27) = 1.72, *p* = 0.201, η_*p*_^2^ = 0.06. The interaction between these two factors was not significant: *F* (1, 27) = 3.6, *p =* 0.07, η_*p*_^2^ = 0.12. However, planned comparisons revealed that for the valuable (Go) stimuli only, participants reported stronger automaticity for long-trained stimuli (mean 4.50, SD 1.46) relative to short-trained stimuli (mean 4.35, SD 1.42),*t* (27) = 2.2, *p =* 0.04, *d*_*z*_ = 0.41. Therefore, self-reported automaticity of responding for valuable outcomes increased with behavioral repetition. For the stimuli signaling not-valuable outcomes there was no significant difference between self-reported automaticity of the NoGo response for long-trained stimuli (mean 4.22, SD 1.34) relative to short-trained stimuli (mean 4.26, SD 1.33), *t*(27) = 0.5, *p =* 0.59, *d*_*z*_ = 0.09. Therefore, behavioral repetition of not responding failed to increase self-reported automaticity.

To investigate the relationship between test performance on value-incongruent test trials and self-reported automaticity of Go and NoGo responding, we correlated SRBAI scores for short- and long-trained valuable stimuli with subsequent test accuracy (alpha adjusted for multiple comparisons to ∝ = 0.0125 using Bonferroni correction). To this end, we calculated difference scores of accuracy on still-not-valuable minus devalued and of accuracy on still-valuable minus upvalued, separately for short- and long-trained stimuli. There were no significant relationships between self-reported automaticity for Go responding and performance on still-not-valuable trials relative to devalued trials (for either short-trained stimuli, *r*(26) = – .013, *p* = 0.948, or long-trained stimuli, *r*(26) = – .004, *p* = 0.985). Similarly, self-reported automaticity for NoGo responding and accuracy on still-valuable relative to upvalued trials was not significant (for either long-trained stimuli, *r*(26) = 0.085, *p =* 0.667, or short-trained stimuli, *r*(26) = 0.333, *p =* 0.08).

#### Working memory

**Fig. 3:**
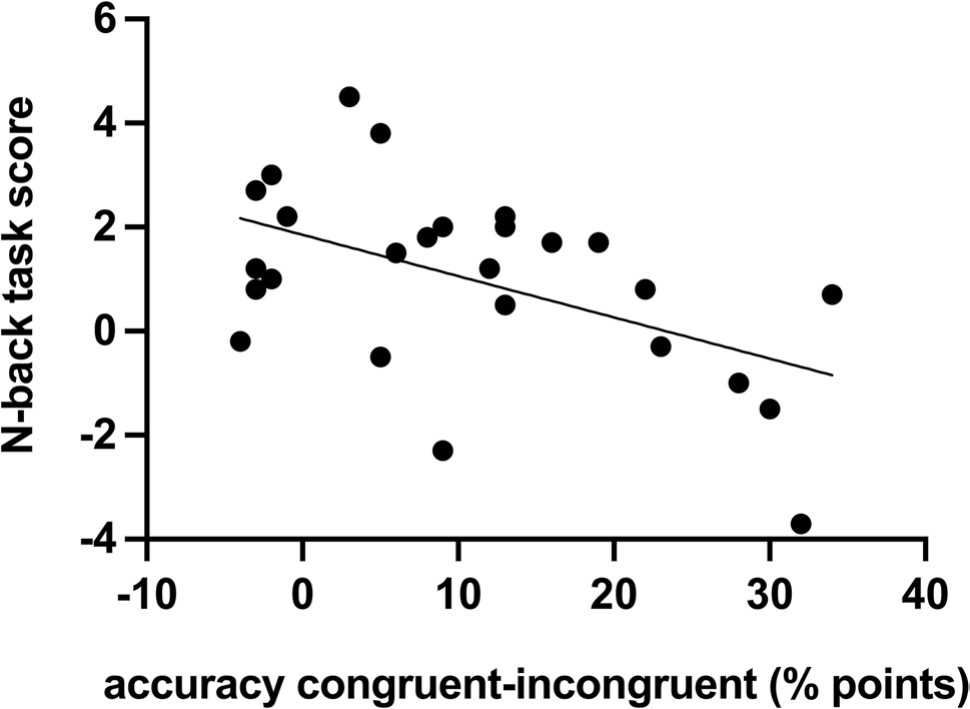
Participants with lower working memory scores (as measured with the N-back task) showed increased deficit in performance on incongruent test trials (relative to congruent trials). This graph has non-zero origin

Two participants did not complete the N-back task. The mean score was 0.99 (SD 1.82). To control for baseline performance, we calculated the accuracy difference for value-congruent trials (mean of still-valuable and still-not-valuable) minus incongruent trials (mean of devalued and upvalued). Participants with better working memory scores had smaller accuracy difference scores indicating better behavioral adjustment after a change in outcome value, *r*(24) = – .513, *p =* 0.007 Fig. 3.

To explore the relationship between working memory and Go versus NoGo test performance trials, we correlated N-back scores to the difference scores for accuracy on still-valuable minus upvalued, and accuracy on still-not-valuable minus devalued, respectively. These analyses revealed that participants with lower working memory scores had a greater advantage on the still-valuable relative to upvalued trials, *r*(24) = – .56, *p* = 0.002. This may reflect that they benefitted more from S-R associations when retrieving the correct response on still-valuable trials compared to upvalued trials where they had to initiate a goal-directed response (when that particular stimulus had never been paired with a Go response during training). The corresponding correlation between habit disadvantage on NoGo devalued relative to still-not-valuable, was in the same direction but failed to reach significance, *r*(24) = – .28, *p* = 0.17. Follow-up statistical comparison of these correlations using the Pearson and Filon (1898) method (as implemented in the cocor R package; Diedenhofen & Musch, 2015), demonstrated that these correlations did not differ significantly from one another (*z* = 1.78, *p* = 0.075).

### Discussion **e**xperiment 1

Humans are generally good at performing in a goal-directed manner, which was reflected in overall high accuracy levels during the symmetrical outcome-revaluation test phase. However, in support of the formation of behaviorally autonomous habits, accuracy was poorer when a change in outcome value required a corresponding change in behavior. This was due to participants responding more in the presence of stimuli that predicted devalued outcomes than to stimuli that predicted still-not-valuable outcomes (i.e., commission errors). Conversely, they were less likely to miss the opportunity to respond and collect points when the signaled outcome was still valuable relative to upvalued (i.e., consistent stimulus-Go response associations decreased the chance of an omission error). These impairments were observed despite excellent explicit knowledge of the outcome available on each trial. Therefore, the symmetrical outcome-revaluation task provides a promising tool to study stimulus-driven, habitual control in humans.

We also included a within-subject manipulation such that during training, half of the stimuli were seen three times more often (long trained) than the other half (short trained). We expected that this extended training would lead to stronger S-R links, in turn leading to more erroneous responding in the test phase in the presence of long-trained stimuli that signaled devalued outcomes, relative to short-trained stimuli. However, we did not find any evidence for differences in test performance as a function of training length. A possible explanation is that the repetition difference between the long and short-trained stimuli was not extreme enough to reveal habit formation. We did find effects of training length on self-reported automaticity of Go (but not NoGo) responses. Specifically, participants reported numerically small but significantly stronger automaticity of (Go) responding to long-trained relative to short-trained stimuli signaling valuable outcomes. We did not find any evidence however, that self-reported automaticity for making responses to stimuli signaling valuable outcomes during training was related to subsequent test performance when those were no longer valuable. There was also no significant relationship between self-reported automaticity for NoGo responding and performance on the still-valuable relative to the upvalued test trials. We note however that this correlation was relatively strong (approx. *r =* .35) suggesting that with larger sample sizes evidence for a relationship between generating a response on upvalued trials and NoGo automaticity during training could potentially be uncovered.

Finally, we examined individual differences in working memory (as measured with the Nback task). We found that working memory capacity was positively related to the ability to flexibly adjust behavior on incongruent trials (after controlling for overall accuracy on congruent trials), potentially because working memory plays an important role in keeping goals actively in mind to guide action selection, thereby overriding habits.

To conclude, while inferior performance on the devalued relative to still-not-valuable trials and better performance on still-valuable relative to upvalued provide evidence for S-R habit formation, we did not observe an influence of training amount on behavioral flexibility and only marginal effects on self-reported automaticity (SRBAI) scores. In Experiment 2, we extended the training across an entire week, and we changed the stimulus distribution so that the long-trained stimuli were seen seven (rather than just three) times more frequently than the short-trained stimuli. We expected that for stimuli with longer training (518 repetitions), subjects would develop stronger habits than for short-trained stimuli (74 repetitions), and that this would be reflected in higher accuracy on both still-not-valuable (relative to devalued) and still-valuable (relative to upvalued) trials, as well as a strong(er) increase in self-reported automaticity.

## Experiment 2

Experiment 2 was similar to Experiment 1, apart from the following important procedural differences. Participants came to the lab on day 1 and were introduced to the training phase of the symmetrical outcome-revaluation task. They were then asked to train at home (30 min each day) for six consecutive days (days 2–7). On day 8 they returned to the lab for a final bout of training and the test phase. During the week-long training, short-trained stimuli were presented 74 times, and long-trained stimuli 518 times. To increase motivation, participants were informed that the points that they earned (across both training and test) would be converted into a cash bonus (up to €5) at the end of the experiment. Finally, we again included the SRBAI to investigate the relationship between participants’ self-reported automaticity and their habitual behavior as measured with the outcome-revaluation task.

### Methods

#### Participants

Based on the number of performance exclusions in Experiment 1 and assuming that a substantial number of individuals would drop out of the week-long study, we aimed to test at least 50 participants in a fixed amount of time (pre-agreed based on experimenter and lab-space availability). Across a 2-month period, 50 participants were recruited from the University of Amsterdam participant website. Advertisements stated that this was a training study where participants would have to install software on their laptop and train at home each day for 30 min (in addition to two lab visits). Participants received either €50 or course participation credits for taking part and a performance-based bonus. Eleven participants were excluded from the final analysis: three dropped out and one participant incorrectly trained at home with the wrong participant number (meaning that the counterbalancing was incorrect). One participant admitted to using a cheat sheet to remember the S-O relationships. Six others responded on less than 25% of upvalued trials during the test phase and were thus excluded for poor performance. The remaining 39 participants (28 females) had a mean age of 23.3 years (SD 6.3 years). Using G*power (Faul et al., 2007) a sensitivity analysis for repeated measures ANOVA of the crucial test phase data (eight trial types, with mean correlation between measures of 0.28) with alpha of 0.05, power of 0.8 and total sample size of 39 indicated sufficient power to detect a small-medium effect (*f* = 0.18). The Psychology Ethics Committee of the University of Amsterdam approved the study.

#### Stimuli and materials

##### Symmetrical outcome-revaluation task (SORT)

The computerized paradigm as outlined in Experiment 1 was used, with two minor differences. The first was that in each block of 16 training trials the two short-trained stimuli were each seen once and the two long-trained stimuli each seen seven times. In addition, we added an *outcome-value memory test* to the end of each test block. Here, participants were shown the four ice creams (randomized order) and using the mouse, they had to select which of the two ice creams had been valuable in the block that they had just completed. No feedback was given. The SRBAI was also used, exactly as outlined in Experiment 1.

#### Procedure

The study consisted of three phases:

##### Day 1: Initial lab visit [60-min session]

The trial version of the Presentation software was installed on the participants’ personal laptops and a modified version of the *symmetrical outcome-revaluation task* was installed by the experimenter (no demo phase, 20 blocks total per run of the task – ten blocks each of van stimuli and scooter stimuli, alternating). They then began the instrumental training phase of the task. Half of the stimuli were trained seven times more frequently than the other half of the stimuli.

##### Days 2–7: Instrumental training at home [6 x 30-min sessions]

Participants continued with the instrumental training at home. They were instructed to train every day for six consecutive days (20 blocks per day – ten blocks each of vans and scooters). They were instructed to try and train at the same time each day and in the same room, with minimal distractions. They were required to send the log files at the end of each day to the experimenter, who logged them and contacted participants who had not sent theirs in.

##### Day 8: Lab visit: Training and test phase in extinction [60-min session]

Participants came to the lab and completed eight final training blocks on their laptop. This meant that across eight days, participants completed 148 blocks (74 of van stimuli and 74 of scooter stimuli) of 16 trials each. They saw the four short-trained stimuli 74 times and the four long-trained stimuli 518 times. They then filled in the SRBAI on another computer before continuing with the test phase. The test phase was exactly as outlined in Experiment 1 (eight blocks; 256 trials in total), with the addition of the outcome-value memory test at the end of each test block. Participants then completed the final test of S-O knowledge.

Participants then completed the Personal Need for Structure Questionnaire (Neuberg & Newsom, 1993), which was included for teaching (master thesis) purposes only and was not analyzed for the present paper. Participants were also asked some questions as to whether they had cheated in any way during the home training.

#### Statistical analysis

We followed the same statistical protocol as outlined for Experiment 1 with the exception that the training data was collapsed across days rather than blocks.

Results of the 39 participants: 12 had completed either too few or too many training trials. Of these, six participants missed a day of training; meaning that they performed 128 blocks in total. Five other participants had issues with their laptops while running the task at home (e.g., running out of batteries, crashing, or sound not being on). Two of these participants did not restart the training that day meaning that they missed approximately ten blocks of training. Three of these participants did restart and begin the training again (all had performed less than one block of extra trials). One additional participant completed an entire 20 blocks of training prior to coming to the final lab session on day 8. We decided not to exclude these 12 participants from the test phase data, but we did perform control analyses to ensure that their inclusion did not change the pattern of results. The eight participants who missed one day of training were not included in the training ANOVA and follow-up analyses due to missing data.

#### Training phase

**Fig. 4:**
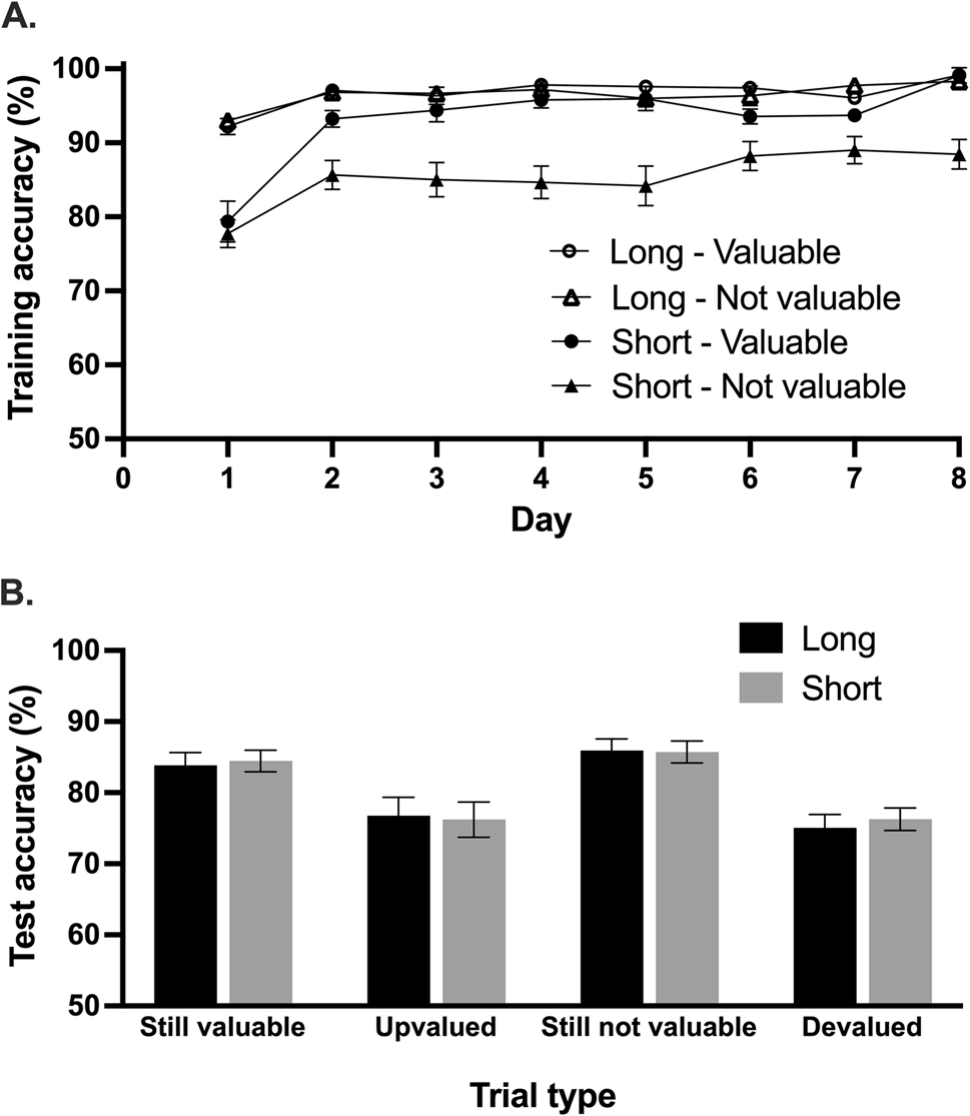
**A** Accuracy in the training phase: Participants learned across the 8 days of training to respond for stimuli that signaled valuable outcomes and to withhold responses for not valuable stimuli. During the final day of training (just prior to the test phase), participants were significantly less accurate for short-trained stimuli that signaled not-valuable outcomes relative to the long-trained stimuli that signaled not-valuable outcomes. **B** Accuracy in the test phase: Participants were less accurate on devalued relative to still-not-valuable trials and more accurate on still-valuable relative to upvalued. There was no significant difference in accuracy on short- relative to long-trained stimuli for any trial type. *Error bars* represent within-subject standard error of the mean (Cousineau, 2005) with Morey correction (Morey, 2008). These graphs have non-zero origins

When examining accuracy as a function of day (1–8), training length (long, short) and value (valuable, not-valuable) a significant three-way interaction between these variables, *F*(7, 210) = 2.70, *p* = 0.034, η_*p*_^2^ = 0.08, was found. As can be seen in Fig. 4, acquisition was superior for long-trained stimuli relative to short-trained stimuli, and this effect of training length was particularly pronounced for the stimuli signaling not-valuable outcomes. This was confirmed by separate analyses of the valuable and not-valuable trials. For valuable trials, the interaction between training length and day was significant, *F*(7, 210) = 12.81, *p* < 0.001, η_*p*_^2^ = 0.30, indicating faster acquisition of the long-trained responses. However, by the final day of training, this difference was no longer significant, *t*(30) = 0.28, *p =* 0.778, *d*_*z*_ = 0.04. For the not-valuable trials there was a significant main effect of training length, *F*(7, 210) = 41.6, *p <* 0.001*, η*_*p*_^*2*^ = 0.58 and a marginal interaction between training length and day, *F*(7, 210) = 2.27, *p =* 0.056, *η*_*p*_^*2*^ = 0.07. Significant differences remained between the short- and long trained conditions on day 8 (just prior to the test phase), *t*(30) = 4.671*, p* < 0.001, *d*_*z*_ = 0.84.

#### Test phase

As in Experiment 1, the analysis of test accuracy revealed a significant main effect of congruence such that participants performed better on trials where the signaled outcome value was congruent with that during training, *F*(1,38) = 25.57, *p <* 0.001, η_*p*_^2^ = 0.40. However, once again, there was no significant main effect of long/short training on accuracy rates in the test phase, *F*<1, *p =* 0.792, η_*p*_^*2*^ = 0.002. The interaction between congruence and long/short training was also not significant, *F*<1, *p =* 0.954, *η*_*p*_^*2*^ = 0.00. Finally, there were no significant effects involving value, *Fs*<1, *p* > 0.332, η_*p*_^*2*^ <0.025 .

As in Experiment 1 we calculated difference scores to examine mean response rates on value-incongruent trials (separately for devalued and upvalued), while controlling for response rates on the value-congruent trials (still-not-valuable and still-valuable, respectively). As in Experiment 1, these scores correlated, *r*(37) = 0.41, *p =* 0.032, indicating that participants who were less adept at flexibly inhibiting responding for stimuli signaling devalued outcomes also had a greater advantage (responding more) on still-valuable relative to upvalued outcomes.

#### Test phase - control analysis

After exclusion of the 12 participants who had received either slightly less or slightly more training than the other participants, the analysis of accuracy during the test phase (collapsed across all blocks) was repeated. The main effect of congruence was still significant, *F* (1,26) = 14.72, *p* < 0.001, η_*p*_^2^ = 0.36. There were no further significant results, all *F*s < 3.58, *p*s > 0.070, η_*p*_^2^< 0.121.

#### Outcome-value memory test

After every block in the test phase, participants were asked to report which two ice creams had been valuable in the previous block. Overall accuracy was high (98%, SD 4%) indicating that participants had retained explicit knowledge of which ice creams they should respond for, throughout the test blocks.

#### Final S-O test

Final S-O test data were missing from three participants. Overall participants had excellent recall of the various S-O relationships after the test phase. Mean accuracy (SD) was 99% (8%) for long-trained and 100% (0%) for short-trained valuable (Go) trials, and 100% (0%) for long-trained and 99% (8%) for short-trained not valuable NoGo trials. Confidence was high overall (mean 99%, SD 2.7%).

#### SRBAI

**Fig. 5:**
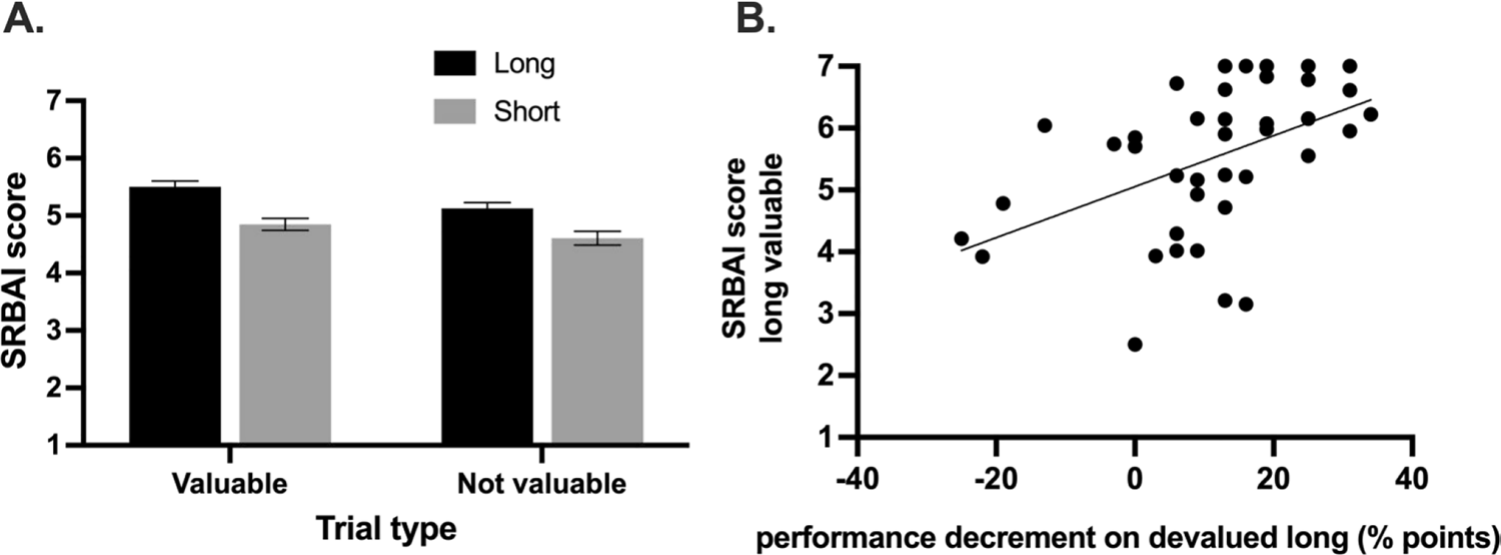
**A** Self-reported automaticity was significantly higher following long training compared to short. **B** Participants who reported stronger self-reported automaticity for long-trained (Go) responses to stimuli that signaled valuable outcomes, made more erroneous slips of action when those outcomes were devalued during the test phase (relative to performance on the still-not-valuable trials). *Error bars* represent within-subject standard error of the mean (Cousineau, 2005) with Morey correction (Morey, 2008). The axes have a non-zero origin

When examining the total score on the SRBAI as a function of training length and outcome value during training (see Fig. 5), we observed a main effect of training length, *F*(1, 38) = 15.9, *p* < 0.001, η_*p*_^2^ = 0.30, such that participants reported stronger automaticity for long-trained stimuli (M 5.3, SD 1.2) relative to short-trained stimuli (M 4.8, SD 1.2). There was also a main effect of value, *F*(1, 38) = 13.3, *p =* 0.001, η_*p*_^2^ = 0.26. Participants reported stronger automaticity when responding for stimuli that signaled valuable outcomes (M 5.2, SD 1.2) versus not responding for not-valuable outcomes (M 4.9, SD 1.1). The interaction was not significant, *F* < 1, *p =* 0.40.

As for Experiment 1, we performed correlational analyses for the devalued and upvalued scores, after controlling for performance on congruent NoGo and Go trials (alpha adjusted for multiple comparisons to ∝ = 0.013 using Bonferroni correction). Firstly, for both long- and short-trained stimuli we correlated SRBAI scores for valuable (Go) stimuli with the accuracy on still-not-valuable test trials minus accuracy on devalued test trials (i.e., action slips). This revealed that participants who reported stronger automaticity for long-trained valuable stimuli performed worse on devalued trials relative to still-not-valuable trials, *r*(37) = 0.477, *p* = 0.002; see Fig. 5B. There was no significant relationship between short-trained valuable SRBAI scores and accuracy on short-trained devalued test trials (relative to still-not-valuable), *r*(37) = –.131, *p =* 0.426. Follow-up statistical comparison of these correlations using thePearson and Filon (1898) method (as implemented in the cocor R package; Diedenhofen & Musch, 2015), demonstrated that the SRBAI-performance correlation for the long-trained condition was significantly stronger than the correlation for the short-trained condition (*z* = 2.95, *p* = 0.002).

Finally, there was no significant relationship between self-reported automaticity for NoGo responding and accuracy on still-valuable relative to upvalued trials, for either long-trained stimuli, *r*(37) = – .050, *p =* 0.761, or short-trained stimuli, *rho*(37) = .209, *p =* 0.201.

### Discussion experiment 2

We replicated the pattern observed in Experiment 1, with superior performance on still-not-valuable relative to devalued trials and still-valuable relative to upvalued trials. This pattern suggests that participants learned to gradually make responses in the presence of discriminative stimuli, leading to rigidity of Go responding, as reflected in commission errors on devalued trials (relative to still-not-valuable) and more regular responding on still-valuable trials (relative to upvalued).

Furthermore, we observed reliable performance differences at the end of training between valuable (Go) relative to not-valuable (NoGo) trials (see Experiment 1 for a non-significant trend in the same direction). In Experiment 2, participants were still responding significantly more for not-valuable outcomes in the short-trained relative to long-trained conditions at the end of training. Participants also reported less automaticity for the stimuli associated with a NoGo response than a Go response. This pattern of results could be related to the task design, which may have enhanced the risk of premature, impulsive (Go) responding because of the fast-moving vehicle and time pressure applied during training. Alternatively, learning in the context of Go and NoGo responses is fundamentally different – a point we return to in the general discussion.

As expected, given the extensive 7-day training protocol, self-reported automaticity (as measured with the SRBAI) was significantly higher for long-trained responses (518 repetitions) relative to short-trained responses (74 repetitions) across both Go and NoGo stimuli. Furthermore, the degree of self-reported automaticity on long-trained trials correlated with the slips-of-action score on devalued long-trained trials during the test phase. That is – participants who reported carrying out long-trained valuable Go responses with more automaticity, found it harder to inhibit this response when the signaled outcome was no longer valuable during test. This was not the case for the slips of action made on short-trained devalued trials. These results suggest that – at least with extended training – participants’ insight into the degree of automaticity with which they carried out a behavior was related to the degree to which habitual responding dominated when they were under time pressure to respond, and the associated outcome had been reduced in value. Furthermore, it suggests that not only Go responses, but also NoGo responses were to some extent subject to habit formation.

In Experiment 2, we trained participants during a week, instead of on a single day (Experiment 1), and we changed the short:long training ratio to 1:7 instead of 1:3. However, we still did not see evidence of increased habitual responding to long-trained relative to short-trained devalued stimuli during the test phase. Nor did the extended training in Experiment 2 appear to impair performance overall compared with Experiment 1. This raises the question of whether variations in the strength of habit formation as a function of behavioral repetition can be captured by experimental outcome-revaluation paradigms. We will return to this issue in the general discussion.

## General discussion

In the present study we used a novel outcome-revaluation task to investigate the balance between habitual and goal-directed control. The symmetrical design of this task allowed us to experimentally demonstrate habitual behavior: participants responded more during devalued trials relative to still-not-valuable (i.e., lower accuracy); and more during still-valuable than during upvalued trials (i.e., higher accuracy). Importantly, these effects were observed despite excellent explicit knowledge of the S/R-outcome relations, suggesting that failures to flexibly adjust responding on the value-incongruent trials were not due to impaired knowledge of the signaled outcome but more likely to the rigidity of S-R associations. Furthermore, those who were less adept at flexibly suppressing responding to stimuli signaling devalued outcomes, also benefitted most from associating stimuli with a Go response when the outcome was still valuable, as reflected in higher response rates towards still-valuable outcomes than to upvalued outcomes. Therefore, participants whose performance suffered most from habit formation following extended repetition of Go responding, at the same time also appeared to benefit most from this behavioral repetition. These consistent patterns across Experiments 1 and 2 suggest that the symmetrical outcome-revaluation task is a useful addition to experimental paradigms currently available to study the ability to adjust responding on the basis of a change in outcome value. Next, we discuss the specific strengths of this task compared to the ‘slips of action’ task used in previous research.

Although similar to the original slips-of-action task (de Wit et al., 2012; Gillan et al., 2011) the symmetrical outcome-revaluation task has a number of important advantages. In the slips-of-action task, participants do not need to pay attention to the outcomes to perform well during the initial, instrumental learning phase. The specific (fruit) outcome identity is irrelevant, because all outcomes are worth points (De Houwer et al., 2018). By contrast, in the symmetrical outcome-revaluation task, only half of the outcomes are worth points during each block of training. This means that participants need to choose to respond to certain stimuli on the basis of the current value of the available outcome. In other words, their responses are mediated by anticipation and evaluation of the outcome, at least initially. In this way, the symmetrical task is better than the slips-of-action task at modelling real-life goal pursuit, as this typically starts out as goal-directed behavior which then potentially transitions to habits (Adams, 1982; de Wit & Dickinson, 2009). Importantly, our results show that despite participants having excellent explicit knowledge of the contingency between stimuli and outcomes and current outcome value, they still made more errors on value-incongruent trials than on value-congruent trials. These results are most readily explained in terms of S-R associations interfering with performance on devalued trials (whilst supporting performance on still-valuable trials). Furthermore, the symmetrical design of the novel task presented in these experiments allows for the critical test comparisons unconfounded by the Go/NoGo nature of the correct response: accuracy on devalued trials is compared with still-not-valuable trials; and accuracy on upvalued trials is compared with still-valuable trials. It should be noted that collapsing across these metrics is not advisable because the speeded task puts different constraints on Go vs. NoGo responses at test (with failures to respond in time registered as a correct response on NoGo trials but an incorrect response on Go trials). The benefit of comparing devalued to still-not-valuable NoGo responses (and still-valuable to upvalued Go responses), is that it permits us to filter out general biases towards Go/NoGo responding. Participants may have higher response rates because they are pressing a key out of fatigue or boredom, or lower response rates due to global slowing causing failures to respond in time. Such differences in baseline rates of responding would be reflected in a main effect of test value but should not skew the (average) comparisons of responding on devalued relative to still-not-valuable and upvalued relative to still-valuable.

The most direct experimental demonstration of S-R habits would be to show that over-trained responses are rendered less sensitive to outcome devaluation relative to more moderately trained responses. However, habitual responding in the current set of experiments was not modulated by behavioral repetition. These results contrast with the animal literature where overtraining does lead to decreased sensitivity to outcome devaluation (Adams, 1982; Thrailkill & Bouton, 2015; although this is not always replicated; Garr et al., 2021). On the other hand, our findings are in line with a previous experimental study in humans (with appetitive and aversive variants of the slips-of-action task as well as an outcome-devaluation task with food rewards), which showed that overtraining did not lead to increased habitual responding (de Wit et al., 2018). There are a number of possible reasons as to why it is difficult to find effects of behavioral repetition on performance with experimental outcome-revaluation paradigms in humans. One explanation is that it simply takes many more behavioral repetitions than are commonly used, across a longer period of time (i.e., weeks or months) to induce a measurable increase in the strength of habits through behavioral repetition. Interestingly, behavioral repetition in our experimental paradigm did lead to increased self-reported automaticity, which in turn was related to habitual slips of action (controlled for baseline response rates for not-valuable outcomes). Furthermore, a related study recently demonstrated that when participants were required to make a new response to a previously (extensively) trained stimulus, error rates did not increase for long-trained stimuli but participants responded slower (potentially reflecting interference of the previously trained response; Luque et al., 2020). It is possible, therefore, that behavioral repetition *does* lead to habit formation but that many more practice trials are required in order to reliably observe a difference in performance (i.e., commission errors on devalued trials). In line with this possibility, it is noticeable that training performance did not reach 100% accuracy until near the end of training and for some trial types, not at all. Furthermore, final SRBAI levels were still far below the maximum score.

Another explanation that has previously been put forward is that the impulse to perform a habitual action may develop relatively quickly, but that humans are very capable of inhibiting these impulses (e.g., Gardner, 2015; Hardwick et al., 2019). Goal-directed control processes may readily override habits, particularly in situations where the context has changed (e.g., when one transitions from training to test; de Wit et al., 2018; Watson & de Wit, 2018). One recent study reported that slips of action (following response-outcome contingency degradation) only emerged in the 300–600-ms range but that at longer response preparation times participants were able to override prepotent responses following extensive training (Hardwick et al., 2019). In support of competition between executive control processes and habitual control, a recent study found that poorer performance on the slips-of-action task was related to increased self-reported attentional demands when overriding a real-life behavior (using a new front door key; Linnebank & Kindt, 2018). In line with this, in Experiment 1 we found that individuals with higher working-memory capacity were better able to flexibly adjust responding following changes in outcome value. This suggests that manipulations that load working memory may allow us to uncover more direct evidence of S-R habits (e.g., combining an overtraining manipulation with a working-memory manipulation during the test phase).

The outcome-revaluation paradigm and self-report measures have both been used extensively to investigate habits, in the respective fields of experimental, and health and social psychology. Here, we investigated the relationship between sensitivity to outcome revaluation and subjective automaticity (e.g., “I do it without thinking”). Across two experiments, we observed that behavioral repetition (S:R→O) led to self-reported stimulus-response automaticity. Furthermore, in Experiment 2, participants with high subjective automaticity for long-trained Go responses, subsequently found it harder to suppress responding when the associated outcome was no longer valuable. We provide, therefore, the first empirical support that these measures are related. This is encouraging because field studies in ecologically valid settings typically do not allow one to quantify behavior in terms of its sensitivity to outcome devaluation (e.g., revaluing a tooth-brushing outcome and then measuring tooth-brushing behavior in extinction). While measuring habit via self-report may seem problematic (Gardner & Tang, 2014; Hagger et al., 2015; Sniehotta & Presseau, 2012), the current study suggests that self-reported automaticity is related to behavioral flexibility as revealed by a more stringent outcome-revaluation test of S-R habit strength. Therefore, our findings suggest that self-reported measures (particularly the SRBAI) can be used to gain insight into habit formation, particularly when more objective measures are difficult to implement. However, there is a caveat here. Although the concepts of automaticity and S-R habits seem at face value to be related, we cannot rule out that the fluency at which participants can retrieve S-O-R associations (supporting goal-directed control) also contributes to the subjective experience of automaticity. This is the first study to investigate the relationship between self-reported automaticity and performance in outcome-revaluation tasks and future studies should attempt to replicate these findings with larger samples to examine this question with more certainty.

So far, we have interpreted performance on the symmetrical outcome-revaluation task as reflecting habit formation of Go responding. Although the design of the task is symmetrical, we assumed that S-Go and S-NoGo learning would not be. Specifically, we anticipated that S-Go habits would mainly contribute to value-congruence effects in the present task: as an advantage on still-valuable trials relative to upvalued, and as a disadvantage on devalued relative to still-not-valuable. However, S-NoGo habit formation may also have taken place (cf. Jahanshahi et al., 2015; Kühn & Brass, 2010) and may also have contributed to the observed patterns of test performance. Using functional neuroimaging, Kühn and Brass (2010) demonstrated that after a training phase where participants could choose to respond or not respond in order to hear different tones, intentionally not responding activated the associated outcome representation (in the auditory cortex) to the same degree as intentionally responding. However, to the best of our knowledge, there has been no previous examination of habit formation of NoGo responses. Interestingly, in Experiment 2, self-reported automaticity was lower for NoGo responses than for Go responses, even though the differences were numerically small. Still, it remains possible that NoGo responses were also subject to habit formation, thus contributing independently to the observed effect of superior performance on still-valuable relative to upvalued. In future research, to directly determine to what extent Go and NoGo training (and possibly habit formation) independently impacted test performance, our paradigm could be modified to include test trials with baseline stimuli that were not previously associated with a Go or NoGo response (but with equally strong S-O associations).

Relatedly, in addition to S-R associations that may differ in associative strength between Go and NoGo, it is also possible that learning of S-O associations is weaker when the outcomes are not valuable during training. Any such difference in S-O associative learning was not evident given the (near-) perfect S-O knowledge in both Experiment 1 and 2, but it remains possible that there could still be differences in the fluency of recall. To investigate this possibility, future studies could use speeded contingency knowledge tests to achieve higher sensitivity in this regard.

Throughout this paper, we have conceptualized habitual responses as behavior that is triggered via direct S-R associations. However, we should point out that indirect S-O-R associations may also contribute to habitual behavior. Stimulus-bound behavior that relies on intact S-O and O-R (rather than S-R associations), has been investigated with the Pavlovian-to-instrumental transfer (PIT) task (see for reviews: Mahlberg et al., 2019; Watson et al., 2018). In this task, participants learn in separate training phases both S-O relationships (Pavlovian training) and R-O relationships (instrumental training). During the test phase, participants continue to make instrumental responses while the Pavlovian stimuli are occasionally presented. Previous studies (in humans and animals) have shown that Pavlovian stimuli can trigger specific instrumental responses directed towards the outcome currently signaled by the Pavlovian cue (an effect known as outcome-specific PIT). As the Pavlovian stimuli have never been trained with an instrumental response, it is presumed that this influence on responding occurs via an S-O-R chain. Despite the fact that the common outcome representation mediates the relationship between stimulus and response in outcome-specific PIT, many studies have suggested that the behavior is not sensitive to outcome devaluation (Corbit et al., 2007; Holland, 2004; Watson et al., 2018; but see: Mahlberg et al., 2019). This leaves open the possibility that indirect S-O-R associations contributed to failures to adjust behavior when the value of the outcome changed in our current paradigm.

Finally, there are limitations to studying habit formation in human participants that should be acknowledged. We have interpreted performance on this task as being either under goal-directed control (based on anticipation and evaluation of the outcome) or stimulus-driven habits. However, it remains possible that explicit strategies played a role in task performance. During the training phase, a rapid shift from goal-directed towards habitual control would be expected to increase speed and efficiency. To accelerate this shift, participants may have spontaneously formed implementation intentions to mentally form S-R associations (e.g., ‘if I see cue X, then I will perform behavior Y’), and thereby deliberately induce ‘strategic automaticity’ (Gollwitzer, 1993). The question arises whether such a strategy would also reduce flexibility during the subsequent outcome-revaluation test. This question remains to be addressed by future research (but for a relevant discussion of ‘flexible tenacity’ see Legrand et al., 2017).

In conclusion, we experimentally demonstrated habitual behavior: as reflected in inferior performance on value-incongruent relative to value-congruent trials at test. Furthermore, we showed that impaired performance on value-incongruent trials was smaller in individuals with relatively high working memory, suggesting that working memory supported goal-directed control. Using the symmetrical outcome-revaluation paradigm, we also showed that behavioral repetition leads to increased self-reported automaticity. Furthermore, self-reported automaticity of extensively trained Go responses was related to the difficulty in inhibiting that response when the associated outcome was no longer valuable. Overall, the symmetrical outcome-revaluation task provides a promising new tool to investigate the balance between goal-directed and habitual control in humans.
